# Comment on “Hall et al., Topical cannabidiol is well tolerated in individuals with a history of elite physical performance and chronic lower extremity pain”

**DOI:** 10.1186/s42238-023-00206-8

**Published:** 2024-03-18

**Authors:** Luis Vitetta, Jeremy D. Henson

**Affiliations:** 1 Faculty of Medicine and Health, University of Sydney, Sydney, NSW 2006 Australia; 2 Prince of Wales Clinical School, University of NSW, Sydney, NSW 2052 Australia

Dear Editor

Hall et al. report an interesting thoughtful study that was published in the *Journal of Cannabis Research *on a retrospective study with the topical use of cannabidiol (CBD) as the single active, in elite athletes (*n* = 20) that presented with disproportional disabling lower limb injuries. There are key points, however, that were not mentioned in their article that should be underlined and that could further explain the drop-out and favourable results reported. These points may add considerate appreciation to future clinical research with cannabis-based medicines for the management of chronic pain.

In the inclusion and exclusion criteria, Hall et al. reported (Hall et al. 2023) that the athletes, as is customary in clinical trials, were asked to discontinue all non-study treatment modalities that included any pharmacotherapy with opioids, NSAIDs, and acetaminophen. There was no information though as to whether the participants had previously or had recently used cannabis-based medicines to manage their pain, following retirement from their professional chosen sports. This is important, given that there was a 30% drop-out and that the use of cannabis, THC, and CBD by athletes is a common occurrence for the purpose of improving performance and recovery, an increasingly reported outcome, across different sports and levels of competition (Burr et al. 2021; Naik and Trojian 2021). Participant naïveté of cannabis-based medicines use is not uncommon (Vitetta et al. 2022) and could in part explain both the drop-out and positive outcomes observed. Often patients do not have their expectations realised especially with pain symptom improvement and hence tend to cease participating in clinical studies (i.e. drop-outs) opting to revert to standard pharmaco-analgesics. Unmet expectations effects on pain amelioration did not eventuate; and in the subgroup that experienced decreases in pain scores with CBD, seemed excessive without any objective markers in support of the data.

No information on the prescribed proprietary CBD formulation was provided other than the primary active was CBD with the inclusion of lemongrass, ylang ylang, wintergreen, and camphor. Providing the percentage content of camphor as well as that of the other ingredients is of critical importance given that camphor oil is a common ingredient in pain relief in topical analgesic products and lemongrass has anti-inflammatory actions, ylang ylang can be used as a carrier oil and the active ingredient in wintergreen is methyl salicylate a close relative of aspirin. A full disclosure of the formulation should be provided with the relative percentage concentrations. Also, in the drug administration section, the authors allude to the fact that CBD is highly lipophilic and that produces limited skin permeation even with chemical enhancement by essential oils. Therefore, were the extra compounds used as excipients or as adjuncts to CBD? Also, what was the plant origin of the CBD, was it from a *Cannabis sativa* plant extract or *Cannabis indica* plant extract or from hemp? Did the proprietary extract contain only CBD or were other cannabinoids present in minor quantities? Clinical efficacy edicts that researchers should ensure that cannabis-based medicines need to be standardised for the active molecule to be administered, and for a clinical efficacy determination to be made if such a result were to be observed. Further, some aspect of cannabinoid quality control and good manufacturing practice must be provided that avoids variations in dose that may result, this is especially important for cannabis-based medicine extracts for the management of pain. To this last point, it is important given that *n* = 7 participants reported adverse effects (AEs) that the authors attributed to the topical CBD formulation.

In their discussion, we agree with Hall and colleagues (Hall et al. 2023) that there are several limitations that the study outcomes present and indeed caution is advised with interpretation of the data, a justified posit where Hall et al. plausibly suggest that for the management of neuropathic limb pain, topical cannabis-based medicines could include THC for further efficacy. A recent report agrees; the review investigated the safe use of cannabinoids for the management of chronic neuropathic pain presenting an efficacy picture of clinical evidence that very much suggests that when THC alone or in combination with CBD was used there was a better suppression of chronic neuropathic pain than with CBD alone (Bennici et al. 2021). A recent study confirms that impression citing CBD alone was ineffective compared to placebo, concluding that there was no statistical difference in the state of neuropathic pain (Sainsbury et al. 2021). Moreover, Hall and colleagues’ (Hall et al. 2023) reference to nabiximols [a CBD to THC oro-mucosal spray in a ratio of 1:1] that shows promising results in the treatment of neuropathic pain is in our opinion not conclusive. Hall and colleagues support this view by citing a recent meta-analysis that determined that nabiximols was superior to placebo with a small effect size, improving chronic neuropathic pain (Dykukha et al. 2021). On the current overall available data, this impression remains in our view contentious, especially when consideration is given to studies that have reported that nabiximols was not superior to placebo on the primary efficacy endpoint as an adjunct to opioids in managing cancer pain or that the evidence was of poor clinical quality (Lichtman et al. 2018; Fisher et al. 2021). Indeed, larger clinical trials are required to reach definitive conclusions on the efficacy of nabiximols to successfully treat chronic neuropathic pain.

Further, the authors (Hall et al. 2023) refer specifically to neuropathic pain, which by definition is reported to be commonly triggered by an event or injury that evokes pain caused by a lesion or disease of the somatosensory nervous system (Finnerup et al. 2021). Notwithstanding this, it is plausible that the pain mechanism for these athletes could have been a combination of neuropathic and nociceptive pain.

## Data Availability

Not applicable.
